# Comparison of BiliCocoon phototherapy with overhead phototherapy in hyperbilirubinemic neonates. A randomized clinical trial

**DOI:** 10.1038/s41390-024-03692-5

**Published:** 2024-11-03

**Authors:** Mette L. Donneborg, Pernille K. Vandborg, Niels H. Bruun, Lars Bender, Tina Møller, Helle H. Thomsen, Finn Ebbesen

**Affiliations:** 1 Department of Pediatrics, North Denmark Regional Hospital, Hjoerring, Denmark; 2 Centre for Clinical Research, North Denmark Regional Hospital, Hjoerring, Denmark; 3 Department of Clinical Medicine, Aalborg University, Aalborg, Denmark; 4 Department of Pediatrics, Viborg Regional Hospital, Viborg, Denmark; 5 Research Data and Biostatistics, Aalborg University Hospital, Aalborg, Denmark; 6 Department of Pediatrics, Aalborg University Hospital, Aalborg, Denmark; 7 Clinical Nursing Research Unit, Aalborg University Hospital, Aalborg, Denmark

## Abstract

**Background:**

Around 2–6% of term or late preterm neonates receive phototherapy for hyperbilirubinemia. Standard treatment today is overhead phototherapy. A new device has been developed, the BiliCocoon, where the neonates are “wrapped” presumably making them more comfortable. The aim was to compare the efficacy and performance of the BiliCocoon with overhead LED phototherapy.

**Methods:**

A randomized open-label multicenter trial in three Danish neonatal units. Healthy hyperbilirubinemic neonates, gestational age ≥33 weeks and postnatal age 24 h to 14 days were randomized to 24 hours’ of treatment with BiliCocoon or overhead blue LED phototherapy with an equal level of irradiance. A mixed effect model with random effect by center was used to compare the percentage decrease in total serum bilirubin (TSB) between the treatments.

**Results:**

Totally 83 neonates were included. Mean TSB reduction in the BiliCocoon group (*N* = 42), adjusted for baseline TSB, was significantly lower than in the overhead LED group (*N* = 41), 29% vs. 38% (*p*-value < 0.01). Overall difference in temperature by treatment (BiliCocoon vs overhead) was 0.70 [0.37; 1.02] °C, *p*-value < 0.01.

**Conclusion:**

Bilirubin reducing efficacy of BiliCocoon was lower than that of overhead phototherapy, but it was sufficient for nearly all neonates during 24 hours of treatment.

**Impact:**

- The BiliCocoon has a bilirubin reducing efficacy, sufficient for almost all neonates during 24 hours of phototherapy.
- The BiliCocoon does not have an equal bilirubin reducing efficacy as overhead phototherapy.
- The duration of light exposure was longer for the neonates treated in the BiliCocoon.
- A few neonates can be exclusively breastfed in the BiliCocoon throughout the treatment.
- The reason for stopping breastfeeding in the BiliCocoon was most often, that the neonates developed hyperthermia.

## Introduction

Neonatal jaundice is a common condition affecting 80% of neonates during the first week postnatally.^1^ For neonates in need of treatment to avoid kernicterus spectrum disorder, the first-line treatment is phototherapy. 2- to 6% of term or late preterm neonates are treated with phototherapy. The effect of phototherapy depends on the level of irradiance applied, the spectrum of the light, the exposed body surface area, the initial total serum bilirubin concentration (TSB), the duration of phototherapy exposure and the hemoglobin level.^2,3^ The most frequently used phototherapy devices emit blue LED light (light emitting diode) either exposing the neonate from overhead light (conventional or single phototherapy) or light both from overhead and below (double-sided phototherapy), or from a fiberoptic LED pad.^4^ For all of these devices the duration of exposure is interrupted during (breast) feeding of the neonate and as regards overhead phototherapy, parents cannot hold the neonate during treatment.

A newer light device, the BiliCocoon (NeoMedLight, Villeurbanne, France), has been developed consisting of a light source and two light emitting pads, a ventral and a dorsal, in which optical fibers are incorporated, with a disposable cover that wraps the neonate like a sleeping bag. The advertised advantages of the BiliCocoon compared with neoBLUE, overhead LED light are the following: (1) a larger body surface area exposed to light, (2) a higher percentage of time where the neonate is exposed to light, (3) higher efficacy, and (4) the possibility that the neonate remains with their parents during the treatment, leading to improved parent-neonate bonding and ability to (breast)feed the neonate without interrupting the treatment.^5,6^

The objective of the present study was to compare the efficacy and performance of the BiliCocoon compared with standard blue, overhead LED phototherapy as regards: (1) efficiency, measured in decrease of TSB during 24 hours (h) of phototherapy (ΔTSB_0-24_), (2) time of phototherapy exposure during a 24 h study period and (3) (breast)feeding during the treatment. Comparing two different phototherapy devices are challenging, but it is important to compare a new device with the standard treatment.

## Subjects and Methods

### Subjects

This study is a randomized open-label multicenter trial conducted in Denmark from October 1, 2020, to March 31, 2022. Study settings were the Pediatric Departments at The North Denmark Regional Hospital (Hjoerring), Aalborg University Hospital (Aalborg), and Viborg Regional Hospital (Viborg). The inclusion criteria were healthy neonates in need of phototherapy with TSB above the treatment limit according to the Danish National Guideline,^7^ gestational age ≥33 weeks, birth weight ≥1800 *g*, and postnatal age between 24 h and 14 days. Exclusion criteria were rapidly increasing or very high TSB or suspected hemolytic disease requiring a more intensive phototherapy or exchange transfusion according to the guidelines.^7^ Neonates with lower birth weight were not included due to the risk of hypothermia. The study was unblinded due to the nature of the intervention given.

### Randomization and procedure

The parents were informed by the doctors in charge, and they also enrolled and assigned the patients to phototherapy. The neonates were randomized to treatment with either BiliCocoon or overhead phototherapy using numbered sealed opaque envelopes generated by one of the authors in each department. The randomization was balanced in blocks of 4, 6 or 8 numbers. The neonates were only included in the study during their first phototherapy. The treatment duration was 24 h measured objectively. The neonates treated in the BiliCocoon were to receive continuous phototherapy throughout the 24 h, except for changing the neonate’s diaper, while the neonates in the overhead group had time-out’s with an interruption for feeding and changing diapers up to 30 minutes every third hour. Data on the demographic and clinical characteristics of the neonates were registered. Further, data on the need for phototherapy beyond 24 h and rebound hyperbilirubinemia with TSB above treatment level was also included. Unlike with overhead phototherapy, the neonates’ eyes were not protected by a pad during treatment with the BiliCocoon, as they were not exposed to light.

The body temperature was measured rectally 3 times during phototherapy treatment, approximately every eight hours. Hyperthermia was defined as a rectal temperature ≥37.5°C and hypothermia as a temperature ˂36.5°C. The room temperatures were 23 to 25 °C. The neonates were weighed at initiation and termination of phototherapy.

Last, the neonates’ parents filled out questionnaires registrating duration and reason for time-outs from phototherapy.

A total of 85 neonates were randomized, 44 to treatment with the BiliCocoon and 41 to treatment with overhead phototherapy. Two neonates in the BiliCocoon group were withdrawn from the study: one due to non-compliance from the parents and one because the blood test was by mistake first taken after 35 hours. Thus 42 infants received treatment with BiliCocoon and 41 overhead phototherapy.

### Devices, level of irradiance, and body surface area measurements

In the BiliCocoon, optic fibers emit blue LED light with peak emission at 460 nm, bandwidth 20 nm (spectral range at half peak emission), and spectral range 430–490 nm. The overhead phototherapy device used was neoBLUE (Natus Medical, San Carlos, CA) emitting blue LED light with peak emission at 458 nm, bandwidth 20 nm, and spectral range 430–490 nm. The level of irradiance of both devices was measured by a neoBLUE radiometer (Natus Medical, San Carlos, CA), which measures the irradiance in the spectral range 420–500 nm with peak sensitivity at 460 nm. The level of irradiance was measured by the energy of the photons as usual. It would have been preferable to measure the photon flow, but such device was not available and is not used clinically.

The level of irradiance of the BiliCocoon with cover was measured directly on the surface of the pads. Each pad had an area of 20 × 30 cm ~1200 cm^2^ in total. The level of irradiance was measured in 24 fields of 7.5 × 6.7 cm and was adjusted to be 30 µW/cm^2^/nm. The irradiance of the neoBLUE device was measured in a light footprint, 9 cm above the mattress level - corresponding to the average height of a neonate - which had an area of 28×49 cm ~1372 cm^2^. The level of irradiance was measured in 28 equal squares (7 × 7 cm). The mean level of irradiance was calculated for both devices.^8^ The distance from the lamp to the mattress was measured with a wood stick for each neonate and was adjusted for each device, entailing an equal level of irradiance to compare the two devices. The mean measured irradiance of the exposed light was BiliCocoon/overhead light: Hjoerring 30.2/31.4, Aalborg 30.4/30.7 and Viborg 31.1/29.1 µW/cm^2^/nm. The distribution of the irradiance within the exposed area was different for the two devices: homogenous for the BiliCocoon and highest centrally for the overhead light. The level of irradiance of the BiliCocoon decreased slowly during use, approximately 0.7 µW/cm^2^/nm/100 h. Therefore, we adjusted the irradiance during the study at all three investigation sites. Adjustment of the level of irradiance is a feature of the device.

The body surface area of a neonate in m^2^ was calculated by the following formula: the square root of (length (cm) x body weight (kg) divided by 3600).^9^ For a neonate measuring 52 cm and weighing 3.3 kg at birth, the body surface area is 0.2183 m^2^ = 2183 cm^2^. The exposed body surface area of a neonate from the overhead device is 30% of the total body surface area,^8,10^ entailing 2183 cm^2^ x 0.3 = 655 cm^2^. Finally, the “bikini” diaper (20 cm × 5 cm ≈ 100 cm^2^) and eye pads (10 cm^2^) cover approximately 110 cm^2^. Making the exposed area in overhead light in this example 545 cm^2^.

The light exposed body surface area in a BiliCocoon is estimated as the total area of the two pads 1200 cm^2^ minus the 15 cm of the body covered by the diaper (2 × 20 cm x 30 cm – 2 × 20 cm x 15 cm) which makes an area of 600 cm^2^. Entailing, that the light exposed area is estimated a little bigger in the BiliCocoon than in the overhead device.

In the BiliCocoon, the intention is, that the whole body of the neonate is directly surrounded by the bag with the pads being tightly adherent to the neonate’s skin, leaving only a very small space around the neonate’s body for ventilation. But in some places, the BiliCocoon is not that tight adherent to the skin. The cover has flaps that can be opened to allow air circulation to prevent hyperthermia, and in this case, there is a distance between the pads and the skin as well.

At the start of phototherapy, TSB (TSB_0_) and hemoglobin concentration were measured from capillary blood drawn by heel prick. After 24 h of phototherapy, TSB was measured again (TSB_24_). TSB was measured by the routine method used in each department: Cobas 8000 Analyzer (Roche Diagnostics International, Mannheim, Germany)^11^ in Aalborg Hospital and Atellica CH (Siemens Healthcare Diagnostics, Camberley, United Kingdom)^12^ in Hjoerring- and Viborg Hospitals. The hemoglobin concentration was measured by a photometric method using ABL 800 Flex Analyzer (Radiometer, Copenhagen, Denmark).

The hemoglobin concentration was measured because hemoglobin competes with bilirubin for the photons. So, during treatment with standard blue light, the decrease of TSB is related to the hemoglobin concentration.^3^ The reference intervals of hemoglobin day 1 to 3 were 9.1–14.9, day 4 to 7 8.5-14.3 and day 8 to 14 8.0–13.6 mmol/L.

### Data analysis

Data from an earlier study about the decrease in TSB during 24 h of phototherapy with blue LED light (neoBLUE) was used for the sample size calculation.^13^ To be able to detect a clinically relevant difference between the two groups of 6 percentage points in the decline of TSB during phototherapy, at least 36 neonates were required in each group. We chose a significance level of 0.05 and a power of 0.8.

Continuous variables are summarized with means and standard deviations or medians and range (min, max) depending on the distribution. Categorical variables are summarized using counts and percentages. To analyze differences in demographic or clinical characteristics between the two treatments or between the three centers, we used ANOVA for comparing means, Kruskal-Wallis tests for comparing medians, and chi-square tests for categorical variables.

We analyzed the total and the percentage decrease in TSB during 24 h of treatment using a random intercept mixed regression by hospital centers with robust variance estimation. As a sensitivity analysis, we adjusted for the TSB_0_. A multiple linear regression model was used to adjust the effect of the type of phototherapy on ΔTSB_0-24_ (%) for factors known from the literature: birth weight, postnatal age, hemoglobin concentration, type of feeding and TSB_0_.

## Results

**Fig. 1:**
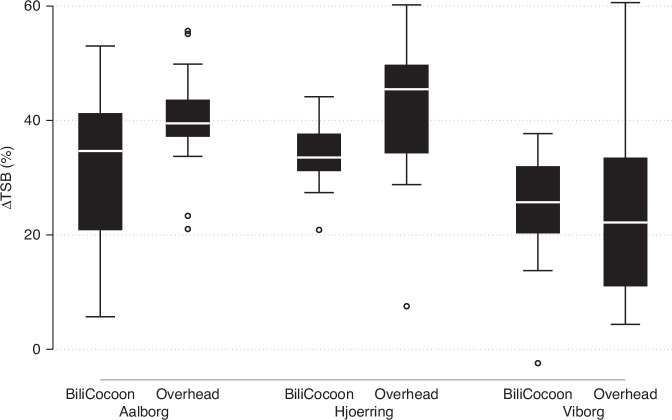
The percentage change in TSB for each group, divided by Center, after 24 h of therapy shown in boxplots.

Baseline demographic and clinical data are shown in Table 1 and the two groups were comparable, no significant differences between the two groups were found. Likewise, the three center populations were similar except regarding postnatal age, where the neonates in Viborg were younger than the ones from Aalborg and Hjoerring. However, no significant differences between the centers were found (see Supplementary Table 1A). The ΔTSB_0-24_ (%) for the BiliCocoon group and the overhead phototherapy group adjusted for TSB_0_ were 29.1% and 37.7%, respectively, the difference was significant (Table 2). After adjustment for confounding variables, the difference was still significant (Table 3). Boxplots show the percentage change in TSB for each group after 24 h of therapy (Fig. 1).

**Table 1:** Demographic and clinical characteristics of the neonates included in the study.

Columns by: Treatment	BiliCocoon	Overhead	Total	*P*-value	Missings/*n* (%)
*n* (%)	42 (50.6)	41 (49.4)	83 (100.0)		0/83 (0.0)
Sex (male), *n* (%)	25 (59.5)	29 (70.7)	54 (65.1)	0.28	0/83 (0.0)
Ancestry (Caucasian), *n* (%)^a^	34 (87.2)	36 (90.0)	70 (88.6)	0.52	9/ 83 (10.8)
Feeding, n (%)				0.35	1/83 (1.2)
*Breastfeeding*	22 (52.4)	19 (47.5)	41 (50.0)		
*Formula*	1 (2.4)	4 (10.0)	5 (6.1)		
*Mixed*	19 (45.2)	17 (42.5)	36 (43.9)		
TSB_0_ (µmol/L), mean (sd)^b^	313 (54)	298 (65)	306 (60)	0.23	0/83 (0.0)
Gestational age (days), median (min; max)	266 (236; 290)	264 (238; 291)	264 (236; 291)	0.28	0/83 (0.0)
Postnatal age at treatment start (h), median (min; max)	97 (25; 265)	93 (30; 231)	93 (25; 265)	0.58	0/83 (0)
Weight loss from birth to start of phototherapy (%), median (min; max)	−5 (−11; 4)	−5 (−13; 3)	−5 (−13; 4)	0.75	1/83 (1.2)
Hemoglobin, mmol/L, mean (sd)	11.9(1.4)	12.1(1.3)	12.0(1.3)	0.53	1/83 (1.2)
Birthweight (g), mean (sd)	3451 (678)	3211 (650)	3332 (671)	0.10	0/83 (0.0)
Weight at initiation of phototherapy (g), mean (sd)	3286 (625)	3062 (575)	3177 (608)	0.10	1/ 83 (1.2)

^a^Two infants were of Asian ancestry, one in each group and 2 infants were of Middle Eastern ancestry, both in the BiliCocoon group.

^b^TSB_0_: TSB at initiation of phototherapy.

**Table 2:** Levels of total serum bilirubin at 0 h and 24 h, and total and percentage decrease of serum bilirubin concentrations in the two groups after 24 h of phototherapy.

		BiliCocoon: mean	[95%	CI]	Overhead: mean	[95%	CI]	Difference: mean	[95%	CI]	*P*-value
Crude	TSB_0_^a^ (µmol/L)	313.3	298.4	328.3	297.7	282.5	312.8	15.7	−5.7	37.0	0.15
	TSB_24_^b^ (µmol/L)	214.7	199.8	229.7	180.9	165.7	196.1	33.8	12.5	55.1	<0.01
	ΔTSB_0-24_^c^ (µmol/L)	98.6	77.4	119.8	116.8	95.3	138.2	−18.2	48.3	11.9	0.24
	ΔTSB_0-24_^c^ (%)	29.6	22.7	36.5	36.6	29.7	43.5	−7.0	12.3	−1.7	0.01
Adjusted^**d**^	ΔTSB_0-24_^c^(µmol/L)	92.7	79.3	106.0	120.4	106.9	133.8	−27.7	40.9	14.5	<0.01
	ΔTSB_0-24_^c^(%)	29.1	23.8	34.4	37.7	32.3	43.1	-8.6	13.3	−3.9	<0.01

^a^TSB_0_: TSB at the initiation of phototherapy.

^b^TSB_24_: TSB after 24 h of phototherapy.

^c^ΔTSB_0-24_: Decrease of TSB during 24 h of phototherapy.

^d^Adjusted for TSB_0_.

**Table 3:** Multiple linear regression with percentage decrease in total serum bilirubin during 24 h of phototherapy as an outcome.

	Coefficient	[95%	CI]	*P*-value
BiliCocoon	−7.41	−11.05	−3.78	<0.01
Birthweight(g)	−0.01	−0.01	−0.01	<0.01
Postnatal age (h)	0.04	−0.01	0.10	0.11
Hemoglobin (mmol/L)	−0.77	−2.20	0.66	0.29
Feeding				
*Formula*	−2.94	−10.72	4.85	0.46
*Mixed*	2.14	−1.83	6.11	0.29
TSB_0_^a^ (µmol/L)	0.15	0.10	0.20	<0.01

^a^TSB_0_: TSB at initiation of phototherapy.

Characteristics during and after treatment in the two treatment groups are shown in Table 4. Phototherapy treatment could be terminated for all neonates after 24 h of overhead phototherapy and for 40 of the 42 neonates treated with the BiliCocoon. A few of the neonates in both groups needed a secondary treatment with phototherapy due to a rebound of TSB, four (10%) in the BiliCocoon group and two (5%) in the overhead-treated group. Both groups of neonates received phototherapy identical to the first treatment.

**Table 4:** Characteristics during and after treatment in the two treatment groups.

Columns by: Treatment	BiliCocoon	Overhead	Total	*P*-value	Missings/*n* (%)
*n* (%)	42 (50.6)	41 (49.4)	83 (100.0)		0/83 (0.0)
Temperature 1 (°C), median (min; max)	37.1 (36.3; 38.5)	36.8 (36.0; 37.7)	37.0 (36.0; 38.5)	<0.01	1/83 (1.2)
Temperature 2 (°C), median (min; max)	37.3 (36.5; 38.3)	36.7 (36.0; 37.5)	37.1 (36.0; 38.3)	<0.01	3/83 (3.6)
Temperature 3 (°C), median (min; max)	37.4 (36.3; 38.3)	36.9 (36.3; 37.9)	37.1 (36.3; 38.3)	<0.01	14/83 (16.9)
Time without treatment (min), median (min; max)	204.0 (30.0; 495.0)	311.5 (60.0; 638.0)	250.0 (30.0; 638.0)	<0.01	9/83 (10.8)
Number of feedings (breastfeeding) during treatment with BiliCocoon(n), median (iqi)	1.5 (0.0; 5.8)				
Time-outs during treatment, median (min; max)	8 (3; 22)	9 (5; 20)	9 (3; 22)	0.17	5/83 (6.0)
Number of time-outs caused by:					
Breastfeeding without discontent, median (min; max)	1.0 (0.0; 8.0)	1.5 (0.0; 10.0)	1.0 (0.0; 10.0)	0.05	
Breastfeeding with discontent, median (min; max)	0.0 (0.0; 1.0)	0.5 (0.0; 9.0)	0.0 (0.0; 9.0)	0.01	
Breastfeeding, nursing without discontent, median (min; max)	3.5 (0.0; 8.0)	5.0 (0.0; 10.0)	4.0 (0.0; 10.0)	0.05	
Breastfeeding, nursing with discontent, median (min; max)	0.0 (0.0; 3.0)	1.0 (0.0; 5.0)	0.0 (0.0; 5.0)	0.23	
Nursing, median (min; max)	3.0 (0.0; 9.0)	0.0 (0.0; 10.0)	1.0 (0.0; 10.0)	0.01	
Nursing and discontent, median (min; max)	0.0 (0.0; 4.0)	0.0 (0.0; 2.0)	0.0 (0.0; 4.0)	0.64	
Discontent, median (min; max)	0.0 (0.0; 3.0)	1.0 (0.0; 6.0)	0.0 (0.0; 6.0)	0.05	
Other than the above, median (min; max)	0.0 (0.0; 3.0)	0.0 (0.0; 7.0)	0.0 (0.0; 7.0)	0.43	
Treatment finished after 24 h (yes), *n* (%)	40 (95.2)	41 (100.0)	81 (97.6)	0.16	0/83 (0.0)
Rebound hyperbilirubinemia (yes), *n* (%)	4 (9.5)	2 (4.9)	6 (7.2)	0.41	0/83 (0.0)

The number of time-out’s during phototherapy (median [min, max]) was 8 [3,22] for the BiliCocoon group and 9 [5,20] for the overhead treated group; in minutes, the numbers (median [min, max]) were 204 [30, 495] and 312 [60, 638], respectively, this difference was significant (*p* < 0.01). Neonates treated in the BiliCocoon were exposed to light 86% of the 24 h treatment period versus 78% for neonates treated with overhead phototherapy. In Table 4 as well the reasons for time-outs in the two groups are displayed.

Breastfeeding was initiated in 30 of 41 (73%) of the breastfed/mixed-fed neonates in the BiliCocoon group at the start of the treatment, unfortunately, 8 had missing information, and for 6 infants, they could not manage to breastfeed in the BiliCocoon. Only five (12%) were exclusively breastfed in the BiliCocoon throughout the treatment duration of 24 h. These five neonates had a percent decrease in TSB of a mean of 30%. The most common reason for stopping breastfeeding in the BiliCocoon was that the neonates developed hyperthermia.

The median rectal temperature was higher for the BiliCocoon group than for the overhead group, Table 4. The overall difference in temperature by treatment (BiliCocoon vs Overhead) was: 0.70 °C [0.37; 1.02], *p*-value < 0.01. Mild hyperthermia was observed in the BiliCocoon group with a maximum temperature of 38.5 °C. In the overhead-treated group, the maximum measured temperature was 37.9 °C. A total of 26 (62%) of neonates in the BiliCocoon group developed hyperthermia versus 3 (7%) of the neonates treated with overhead phototherapy. On the contrary, 2 neonates (5%) treated with the BiliCocoon developed mild hypothermia (36.3 °C) versus 15 neonates (37%) treated with overhead phototherapy with lowest measured temperature of 36.0 °C.

## Discussion

The overall result of this study showed that TSB decreased significantly less using BiliCocoon than the overhead phototherapy device. Also, that neonates treated with the BiliCocoon had shorter time-out’s. For the BiliCocoon, a longer duration of light exposure and the light exposed body surface area was suggested to be a little greater than for the neonates in the overhead group. Thus, it could be expected that the efficacy would be greatest for the BiliCocoon group, but the opposite was the case. A contribution to this might be a small reduction of the level of irradiance following the BiliCocoon in some places was not quite adherent to the neonatal skin.

In contrast to our result, a few earlier studies have found the BiliCocoon to be equally efficient to other phototherapy devices.^5,14,15^ None of these studies were randomized trials comparing two LED devices, with an equal measured level of irradiance. Luciano et al. ^5^ compared the BiliCocoon with a historical group of neonates, who had received phototherapy using a single fiberoptic pad (BiliSoft, GE Health Care) large enough to be wrapped around the neonate. For both devices, the level of irradiance was about 35 µW/cm^2^/nm, obtained by the technical features.^5^ Montealegre et al. ^14^ conducted a randomized trial comparing the BiliCocoon with a phototherapy device with fluorescent tubes (Medix LU-6T). The measured levels of irradiance for the fluorescent tubes and the BiliCocoon were 37.3 ± 10.3 µW/cm^2^/nm and 36.0 ± 2.6 µW/cm^2^/nm, respectively.^14^ In a non-randomized study Noureldein et al. ^15^ compared the BiliCocoon in a home-care setting with a LED overhead device (Bililux, Dräger, Germany). The level of irradiance was not measured, but they used the manufacturer information of 35 µW/cm^2^/nm for the first device and >33.5 µW/cm^2^/nm at 50 cm distance for the latter.^15^ Coquery et al. ^6^ did not make a randomized trial, but the intention was to prove the use of the BiliCocoon for continued phototherapy at home, and they found the device proper for this kind of treatment. They used it for neonates with a high risk of hyperbilirubinemia and did not measure the level of irradiance, but used the manufacturer information’s of level of irradiance, 35 µW/cm^2^/nm.^6^

The supposed advantages of the BiliCocoon compared with the overhead LED phototherapy device were not proven to be followed by a larger decrease in ΔTSB_0-24_ (%), with an equal level of irradiance, in this trial. But other advantages for the BiliCocoon must be considered. Even though the decrease in TSB was significantly less, all but two of the neonates treated in the BiliCocoon were below the threshold for discontinuing phototherapy after 24 h and with no more rebound in this group. The neonates do not need to be separated from the parents during the treatment, and the parents could hold the neonate during the treatment possibly leading to a better parent-neonate bonding. Also, the mother has the possibility to breastfeed without interrupting the treatment. This has also been found in a study by Ung et al. ^16^ comparing intensive “tunnel” phototherapy for 6 h with phototherapy in the BiliCocoon for 12 h to avoid hospitalizations and failure rate with the BiliCocoon as well as to assess the length of stay. They found the BiliCocoon safe and effective and facilitating less mother-infant separation as well as no difference in the length of stay.^16^

The boxplots and Table 1A showed an inter-institutional variation of device performance. The median ∆TSB_0-24_ was lower for both groups in Viborg Hospital compared with the two other hospitals. The reason for this variation might be that the neonates’ postnatal age was lower in Viborg entailing a lower TSB_0_ and ∆TSB_0-24_ since the bilirubin level would increase physiologically at this early postnatal age. A possible generally lower TSB level in Viborg Hospital does not change the results as ∆TSB_0-24_ (%) is independent of TSB_0_. The two different methods used for the determination of TSB might influence ∆TSB_0-24_ (%), but equally in the two phototherapy groups in each hospital.

Hyperthermia is a point to consider. Many of the neonates receiving phototherapy in the BiliCocoon developed mild hyperthermia, 62% versus 7% in the overhead group. The study by Luciano et al. ^5^ also reported on hyperthermia for neonates treated in the BiliCocoon, they found, that 12% developed hyperthermia during the treatment. Montealegre et al. ^14^ found a significantly higher axillary temperature using the BiliCocoon compared to overhead phototherapy. However, the difference was only 0.2 °C, which was considered to be clinically irrelevant.^14^ The mechanism of hyperthermia might be multifactorial: (1) The blue LED light includes a small fraction of infrared light (heat), which is absorbed in the neonatal skin. (2) Absorption of photons by hemoglobin and bilirubin is among others converted to heat. Aydemir et al. suggested, that the hyperthermia was related to the increased release of pyrogene cytokines such as TNF-α, IL-1, IL-6, and interferons during phototherapy.^17^ (3) During blue LED fiberoptic phototherapy using a single pad, the temperature of the surface of the pad increased. The reason was suggested to be, that the photons were absorbed by the plastic optic fibers and the plastic cover of the pad converting light energy to thermal energy. (4) Using the BiliCocoon, hyperthermia is also related to the close adherence of the pads to the neonates’ skin only allowing little ventilation of the small space around the neonate reducing the conductive heat loss.^5^ Both in the study by Luciano et al. ^5^ and in our study the hyperthermia was treated by loosening the bag and placing the arms outside the BiliCocoon, resulting in greater ventilation around the neonate, the exposed body surface area being less, and the skin area for heat loss outside the BiliCocoon being increased.

The irradiance of the BiliCocoon decreased slowly during the study period. It might be explained by overheating of the light source. Therefore, we needed to adjust the level of irradiance for the BiliCocoon during the study period for all three investigation sites, to fulfill equal level of irradiance for the two devices.

The study had some limitations: The geometry of the two devices as well as the distribution of the light at the exposed areas were different. Also, measurement of the level of irradiance was different, directly on the surface of the pads with cover for the BiliCocoon and at the preferred distance for the overhead device, to obtain the same level of irradiance.

The strengths of the study were: (1) the study population was homogeneous. (2) The quality of the blue LED light (emission peak and bandwidth) and the level of irradiance were equal for the two groups. (3) The level of irradiance was measured by the same radiometer with a maximum sensitivity corresponding to the emission peaks of the light. (4) The study was performed in three departments, which makes the results more generalizable compared with a study performed in one department and sensitivity analyses have been made to adjust for variations between departments.

## Conclusion

Even though the BiliCocoon does not have an equal bilirubin-reducing efficacy as overhead phototherapy and many neonates experience hyperthermia it still has a bilirubin-reducing efficacy that is sufficient for almost all neonates during 24 hours of treatment. A few neonates were exclusively breastfed in the BiliCocoon throughout the treatment. The reason for stopping breastfeeding in the BiliCocoon was most often that the neonate developed hyperthermia.

## Supplementary information

CONSORT 2010 Checklist

CONSORT 2010 Flow Diagram

Supplementary material

## Data Availability

The materials described in the manuscript, including all the raw data, will be freely available to any researcher wishing to use them for non-commercial purposes, without breaching participant confidentiality.
