# Supplementary material for: Comparison of BiliCocoon phototherapy with overhead phototherapy in hyperbilirubinemic neonates. A randomized clinical trial
Source: Pediatr Res. 2024 Nov 3;97(6):1951–7. doi: 10.1038/s41390-024-03692-5 (PMC12122373; doi:10.1038/s41390-024-03692-5)
Supplement: Supplementary file 2 — CONSORT 2010 Flow Diagram [file 41390_2024_3692_MOESM2_ESM.doc]

**
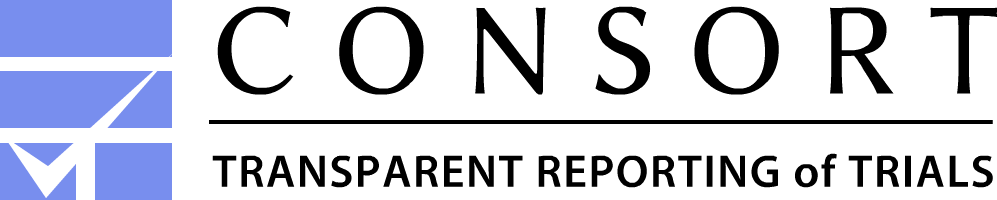
**

**CONSORT 2010 Flow Diagram**

**Allocation**

**Analysis**

**Follow-Up**

**Enrollment**

Assessed for eligibility (n=87)

Excluded (n=2)

♦  Not meeting inclusion criteria (n=2)

Analysed (n=42)
♦ Excluded from analysis (give reasons) (n=0)

Lost to follow-up (n=0)

BiliCocoon group:

Allocated to intervention (n=44)

♦ Received allocated intervention (n=42)

♦ Did not receive allocated intervention (1 due to non-compliance from the parents and 1 because the blood test was by mistake first taken after 35 hours.) (n=2)

Lost to follow-up (n=0)

Overhead group:

Allocated to intervention (n=41)

♦ Received allocated intervention (n=41)

♦ Did not receive allocated intervention (give reasons) (n=0)

Analysed (n=41)
♦ Excluded from analysis (give reasons) (n=0)

Randomized (n=85)
