# Supplementary material for: Comparison of BiliCocoon phototherapy with overhead phototherapy in hyperbilirubinemic neonates. A randomized clinical trial
Source: Pediatr Res. 2024 Nov 3;97(6):1951–7. doi: 10.1038/s41390-024-03692-5 (PMC12122373; doi:10.1038/s41390-024-03692-5)
Supplement: Supplementary file 3 — Supplementary material [file 41390_2024_3692_MOESM3_ESM.pdf]

Supplementary material:

Supplementary Table 1A: Demographic and clinical characteristics of the neonates included in the study, divided by Center.

Columns by: Hospital	Aalborg	Hjørring	Viborg	Total	P-value	Missings / N (Pct)
n (%)	38 (45.8)	27 (32.5)	18 (21.7)	83 (100.0)		0 / 83 (0.0)
Sex (male), n (%)	23 (60.5)	20 (74.1)	11 (61.1)	54 (65.1)	0.49	0 / 83 (0.0)
Etnicity (Caucasian), n (%)	28 (82.4)	24 (88.9)	18 (100.0)	70 (88.6)	0.07	9 / 83 (10.8)
Feeding, n (%)					0.93	1 / 83 (1.2)
Breastfeeding	18 (48.6)	13 (48.1)	10 (55.6)	41 (50.0)		
Formula	3 (8.1)	1 (3.7)	1 (5.6)	5 (6.1)		
Mixed	16 (43.2)	13 (48.1)	7 (38.9)	36 (43.9)		
TSB_0 (µmol/L), mean (sd)[a]	309 (45)	315 (64)	284 (77)	306 (60)	0.21	0 / 83 (0.0)
Gestational age (days), median (min; max)	258 (248; 290)	266 (236; 290)	270 (238; 291)	264 (236; 291)	0.62	0 / 83 (0.0)
Postnatal age at treatment start (hours), median (min; max)	96 (27; 229)	97 (44; 265)	64 (25; 186)	93 (25; 265)	0.15	0 / 83 (7.2)
Weight loss from birth to start of phototherapy (%), median (min; max)	-4 (-9; 2)	-5 (-13; 4)	-5 (-11; 3)	-5 (-13; 4)	0.64	1 / 83 (1.2)
Hemoglobin, mmol/L, mean (sd)	12 (1)	12 (1)	12 (1)	12 (1)	0.24	1 / 83 (1.2)
Birthweight (g), mean (sd)	3282 (636)	3333 (701)	3436 (723)	3332 (671)	0.73	0 / 83 (0.0)
Weight at initiation of phototherapy (g), mean (sd)	3129 (562)	3158 (650)	3312 (653)	3177 (608)	0.58	1 / 83 (1.2)

[a]TSB_0: TSB at initiation of phototherapy
